# Photophysical Properties of the PVK-MEH-PPV/PCBM Composite for Organic Solar Cells Application: Synthesis, Characterization and Computational Study

**DOI:** 10.3390/polym13172902

**Published:** 2021-08-28

**Authors:** Faten Abbassi, Mohamed Mbarek, Maha Almoneef, Kamel Alimi

**Affiliations:** 1 Laboratoire de Recherche: Synthèse Asymétrique et Ingénierie Moléculaires des Matériaux Nouveaux Pour L’électroniques Organiques (LR18ES19) Faculté des Sciences de Monastir, Université de Monastir-Tunisie, Monastir 5000, Tunisia; faten.abbassi@hotmail.fr (F.A.); mohamedmbarek99@yahoo.fr (M.M.); kamealimi@yahoo.fr (K.A.); 2 Physics Department, Faculty of Science, Princess Nourah Bint Abdulrahman University, Riyadh 11594, Saudi Arabia

**Keywords:** composite, materials science, optical properties, DFT calculations, charge transfer

## Abstract

The physical and chemical properties of a new organic composite including PVK-MEH-PPV bi-block copolymer and [6,6]-phenyl-C61-butyric acid methyl ester (PCBM) were recorded. The functionalization and the charge transfer that occurs between donor and acceptor were examined and computed. In fact, the stationary and time-resolved photoluminescence properties were used to examine the effect of the PCBM on the optical properties of the PVK-MEH-PPV matrix. The photoluminescence quenching accompanied by faster PL decay confirmed the charge transfer and interaction process. The electrical and optoelectronic properties and the charge carriers’ injection in the resulting composite were examined. The experimental conclusion was corroborated and confirmed by a calculation based on density functional theory (DFT). Hence, the combination of experimental and theoretical results indicated that the result composite can be applied as an active layer for organic solar cells.

## 1. Introduction

The mastering of coupling optoelectronic properties and polymer structures makes the emergence of organic electronics an interest [1,2,3,4] for scientists hoping to work in this field of research. Their challenge is to at least obtain a stable organic material that is also flexible and can be implemented [5,6,7] as an active layer in optoelectronic devices, especially organic solar cells. Actually, it has been reported that the architectures of the most efficient photovoltaic cells use alternating donor–acceptor groups [8,9,10,11,12,13], mainly for their good thermal stability. In this context, poly[2-methoxy-5-(2-ethylhexyloxy)-1,4-phenylenevinylene] (MEH-PPV) is a semiconducting polymer of interest [14,15,16], one that has been widely studied for the development of a P-N junction with [6,6]-phenyl-C61-butyric acid methyl ester (PCBM) [17,18,19]. However, fullerene and its derivatives, particularly PCBMs, are becoming an interesting candidate for organic optoelectronics thanks to their advantageous properties, mainly their quantum yield at high fluorescence along with their good thermal stability [20,21,22]. In our previous work [23], we already synthesized and studied a new bi-block copolymer including MEH-PPV and PVK. In addition, it is important to note that block copolymers have received strong attention for many years. The photophysical properties of this new copolymer have been investigated by infrared (FT-IR), absorption (UV-Vis), photoluminescence (PL) and time-resolved photoluminescence (TR-PL) spectroscopy and by thermogravimetric analysis (TGA). The resulting copolymer appears to be a good candidate for optoelectronics devices due to its interesting photophysical properties. We are engaged in examining PCBMs (acceptor electrons) based on composites because we are motivated by their good optoelectronic characteristics and seek to improve and strengthen the charge transfer process. The mixture-based copolymer and PCBM can lead to the improvement of charge transport reaching the electrodes and the enhancement of charge collection.

Here, we studied the vibrational and thermal properties of a new composite (copolymer: PCBM) and its optoelectronic properties. A complementary theoretical study of the electronic structure, molecular orbitals and optical absorption of the composite, including the model structure of the previous copolymer [23] and the PCBM, was conducted. The interactions of PCBM and the copolymer matrix (stacking interactions) and their effect were also examined. An energy diagram based on the resulting composite was proposed in which the composite was applied as the active layer and tested with different electrodes. The purpose of this study was to describe the properties of the new composite when the effect of PCBM was added to the photophysical properties of the PVK-MEH-PPV matrix. A combined experimental and theoretical study was used towards a better examination of the properties of the PVK-MEH-PPV/PCBM mixture and their functionalization, as well as the charge transfer process.

## 2. Materials and Methods

### 2.1. Preparation of Composite (PVK-MEH-PPV/PCBM)

The poly [2-methoxy-5-(2-ethylhexyloxy)-1,4-phenylenevinylene] (MEH-PPV) (M_n_ 40,000–70,000), poly(N-vinylcarbazole) (PVK) (M_n_ 25,000–50,000), [6,6]-phenyl-C61-butanoate de methyle (PCBM), ferrichloride (FeCl_3_), chloroform, ethanol and hydrazine used for the synthesis of the composite were purchased from Sigma Aldrich [23]. It is important to recall that we have previously discussed a PVK-MEH-PPV copolymer with 57% polymerization yield. However, the copolymer was insoluble in a common solvent. Therefore, we reconstructed the same route of synthesis [23] by adding the PCBM’s mass in order to obtain a new composite with a 1:1 weight ratio [22]. In more detail, in this method of synthesis we proceeded to the copolymerization reaction of the copolymer with the presence of PCBM in the same solvent. We carried out the copolymerization of the PVK-MEH-PPV copolymer in the chloroform where the PCBM was well-dispersed [22]. The synthesis and the addition of the PCBM were undertaken at room temperature (RT) under an argon atmosphere.

### 2.2. Characterization Methods

The thermal gravimetric analysis (TGA) of the PCBM, copolymer and composite was undertaken with a TGAQ50 (TA Instruments) managed by a heat control program [23]. TGA measurements were recorded under a nitrogen atmosphere between 20 °C and 800 °C. The infrared (IR) spectra were recorded using a Nicolet iS10 infrared spectrometer [23]. A small amount of composite was put on a platinum substrate and then the IR measurements were registered using a diamond connected to the OMNIC software package. NMR spectra were acquired at room temperature (RT) using a Brüker Advance 300 MHz for the spectrometer, operating at 125.7 MHz for ^13^C, using a 4 mm double bearing Brüker probe head [3]. The scanning electron microscopy analysis was carried out using a JEOL 6400F microscope: PVK-MEH-PPV powder was deposited onto a previously cleaned copper pad and covered with a thin, holey carbon film [3,6,22]. The optical absorption measurements were carried out using a Cary 5000 spectrometer at room temperature [3,6,22]. A Jobin Yvon Fluorolog 3 spectrometer with a Xenon lamp (500 W) was used to perform the photoluminescence (PL) measurements [3,6,22]. Time-resolved photoluminescence (TR-PL) was accomplished with a regenerative amplified femtosecond Ti:sapphire laser system (Spectra-Physics Hurricane X). For optical measurements, samples were put on a metal support and covered with a quartz substrate [22].

### 2.3. DFT Calculation

The copolymer and the composite were theoretically studied using the DFT method with nonlocal correlation functional exchange (B3LYP). All these calculations were carried out with the Gaussian 09 software package [24]. The optimization of the composite was achieved with the DFT/6-31G* basic set and time-dependent density functional theory (TD-DFT) was used to predict the optical absorption spectrums of the copolymer and composite. The electronic transition assignments and oscillator strengths of the theoretical optical absorption were simulated using the SWizard program [25].

## 3. Results and Discussion

### 3.1. Infrared Analysis

**Figure 1:**
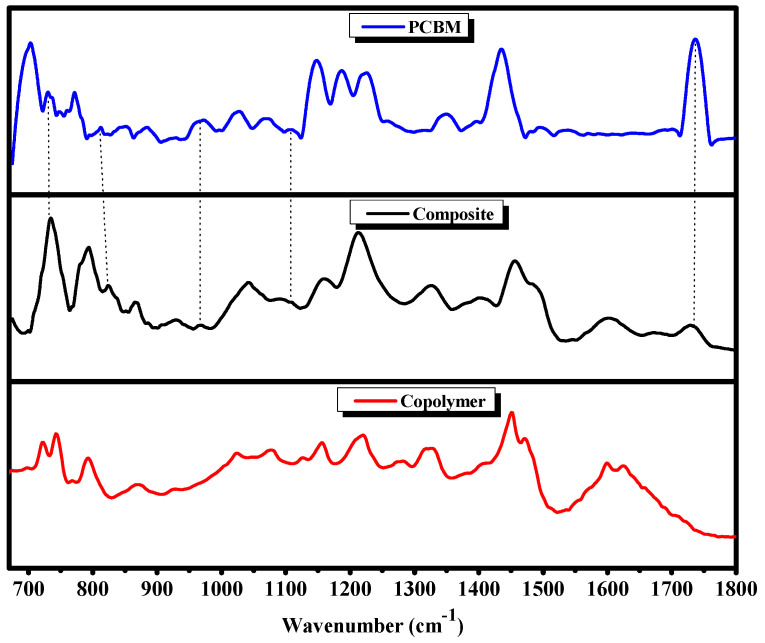
Infrared spectra of copolymer, composite and PCBM.

The composite infrared spectroscopy was carried out by comparing its spectrum with those of the copolymer [20] and the PCBM (Figure 1).

After normalizing the composite and the copolymer IR spectra using the band situated at 1450 cm^−1^, we noticed some modifications in the bands, especially in terms of shape, position and intensity. The two PVK bands [26,27] situated at 720 and 740 cm^−1^, which corresponded respectively to the ring deformation and CH_2_ vibration, were present in the copolymer spectrum but overlapped with the PCBM band situated at 730 cm^−1^ and were molded together only by a band situated at approximately 735 cm^−1^. The same behavior was detected in both PVK bands, which were located at approximately 1600 and 1625 cm^−1^ and which were attributed respectively to stretches of benzene CH_2_ and C-C. These modifications can also be interpreted in terms of the charge transfer between the PCBM and PVK-MEH-PPV matrix and the steric hindrance in the composite caused by the presence of PCBM. The load transfer produced other changes in the intensity band, which were at about 1214 cm^−1^ and 1155 cm^−1^, respectively, and corresponded to the CN stretch and the deformation in the CH plane of the PVK aromatic cycle. The composite (PVK-MEH-PPV:PCBM) was formed by the presence of MEH-PPV bands [22,28] with some changes of position to the 858 cm^−1^ out-of-plane phenyl CH wag and the alkyl-oxygen stretch of 1040 cm^−1^.The intensity ratio (I_composite_/I_copolymer_) of the two bands was calculated to be 0.39 and 1.71, respectively. On the one hand, it is notable that the composite (PVK-MEH-PPV:PCBM) was correctly formed, thanks to the existence of certain MEH-PPV bands [22,28], but with some changes in positions, such as the 858 cm^−1^ out-of-plane phenyl CH wag, the CH wag double-trans band of 969 cm^−1^ and the 1040 cm^−1^ alkyl-oxygen stretch. On the other hand, the intercalation of PCBM in the copolymer matrix resulted in the appearance of certain PCBM bands [29,30,31], such as the C=O band located at 1730 cm^−1^, which had a clear decrease in intensity, as well as several other bands, which are indicated by stars in Figure 1. All these modifications were assigned to an interaction (donor–acceptor) and the charge transfer between the copolymer and PCBM.

### 3.2. ^13^C NMR Analysis

**Figure 2:**
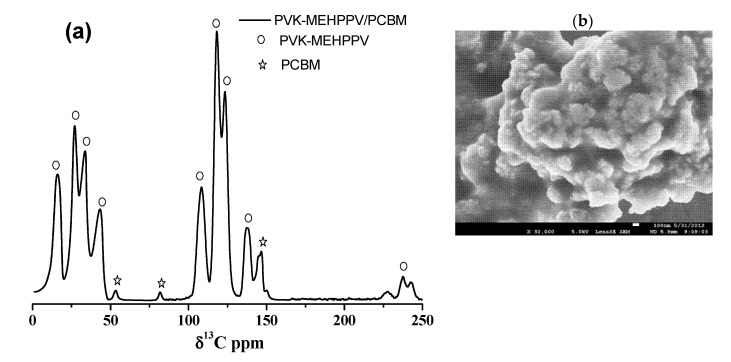
(**a**) ^13^C NMR spectrum of the composite at room temperature; (**b**) SEM image of the copolymer.

Figure 2a was used to assign the ^13^C chemical shifts of the PVK-MEH-PPV/PCBM composite. From this spectrum, the peaks at 16, 27, 34, 43, 108, 118, 123 and 137 ppm were assigned to the chemical shift of PVK-MEH-PPV. The other peaks, marked by stars at 53, 81 and 146 ppm, were attributed to chemical shifts of the PCBM. Figure 2b displays an SEM micrograph of the copolymer. 

### 3.3. Thermal Analysis

**Figure 3:**
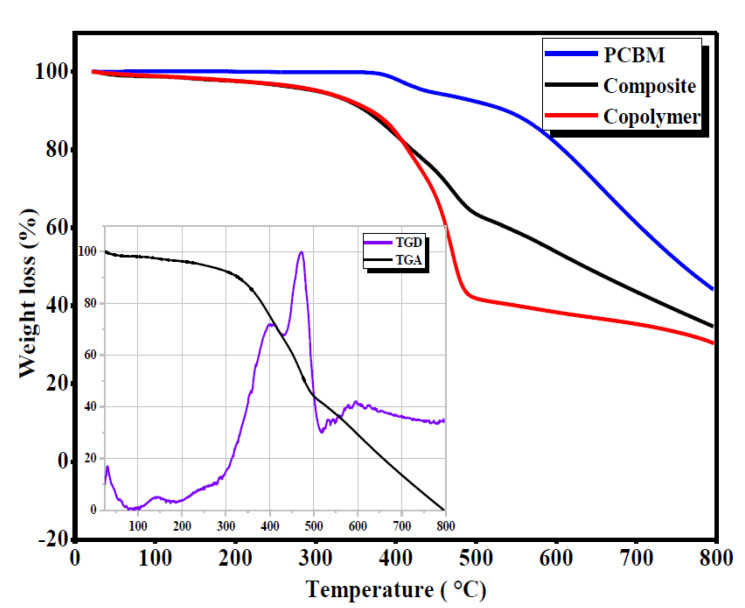
TGA micrograph of the composite, copolymer and PCBM.

Comparing the TGA curves of the PCBM, copolymer and composite (Figure 3), it can be noted that the PCBM exhibited some decomposition located at approximately 400 °C, which was interpreted as the decomposition of phenyl butyric acid methyl ester [20].

The weight loss spectrum of the copolymer showed two steps of decomposition. The first 20% of the decomposition step began at 250 °C and ended at 430 °C, with a maximum observed rate at 420 °C. However, the second 40% decomposition stage occurred between 430 °C and 550 °C, with a maximum rate of mass loss at around 470 °C. Concerning the decomposition of the composite, we noted that its thermal behavior was ameliorated compared to the copolymer alone. Thus, it is clear that the TGA curve of the composite was situated between those of the PCBM and the PVK-MEH-PPV copolymer. From the TGA and the DTG curves of the composite alone (inset Figure 3), we decomposed its degradation into three essential steps. The first, located at 140 °C (3% weight loss), can be interpreted in terms of the evaporation of the solvent used in the synthesis. The second was situated between 280 °C and 425 °C, with a maximum of about 400 °C. The third step was situated between 425 °C and 490 °C, with a maximum situated at 470 °C. We noted that the TGA curve of the composite had the same form as that of the copolymer, with some improvement in terms of weight loss. In summary, adding PCBM to the copolymer matrix enhanced its thermal stability. This is notable for the application of the resulting composite as an active layer.

### 3.4. Optoelectronic Properties

**Figure 4:**
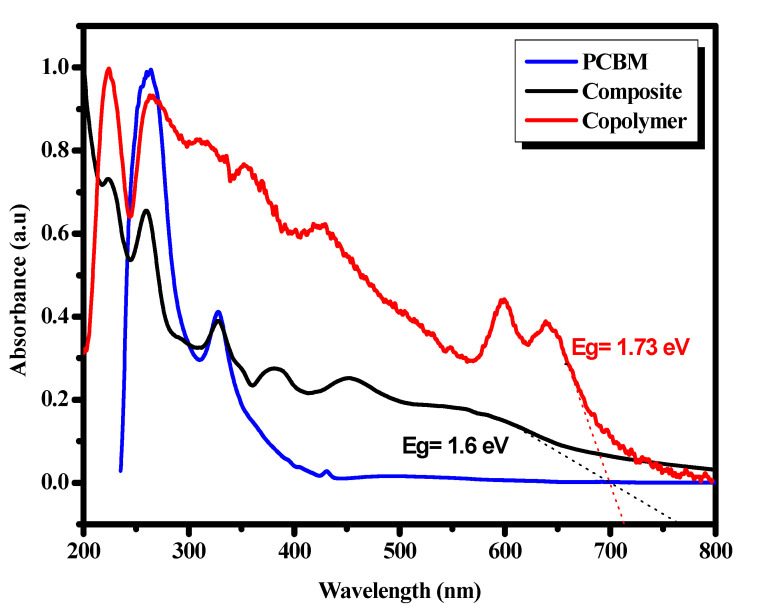
Optical absorption spectra of the copolymer, composite and PCBM.

Figure 4 illustrates the optical absorption spectra of the copolymer [23], the PCBM and the composite recorded at RT.

The data for all compounds were normalized. It is important to remember that the different absorption bands of our previous copolymer [23] were located at 225, 262, 311, 353, 423, 600 and 640 nm. For PCBM, absorption was observed in the near-UV region with two main peaks at 263 and 329 nm, while the spectrum of PVK-MEH-PPV: PCBM showed several sharp bands located at 223, 259, 293, 327, 350, 382 and 454 nm. The presence of a broadband located between 530 nm and 660 nm was noted. These two new bands could be referred to the interband optical transition of the PCBM and confirmed the interaction between the copolymer and PCBM in the backbone of the copolymer skeleton, which supposes a charge transfer. However, after comparing the UV-Vis spectrum of the composite with those of the PCBM and the copolymer, a slight blueshift in the absorption bands of the copolymer was observed, indicating it was mixed with the PCBM, resulting in a decrease in the length of the copolymer. Hence, the conjugation of the copolymer and a new macromolecular arrangement of the copolymer chains appeared. It is important to note the presence of the 329 nm band in the composite spectrum attributed to the PCBM moieties, which signifies the functionalization of PCBM in the PVK-MEH-PPV matrix. Moreover, the two bands of the charge transfer, located at 600 and 640 nm in the copolymer [23] spectrum, were less accentuated in the composite spectrum (the region of 530–660 nm), which indicates the decrease in the organization of the copolymer chains. Subsequently the optical gap of the obtained composite was estimated to be 1.6 eV compared to 1.73 eV for the copolymer alone, and this indicates the good interaction and the charge transfer process in the composite. The resulting low band-gap of the composite seems to be applicable for solar cell uses.

**Figure 5:**
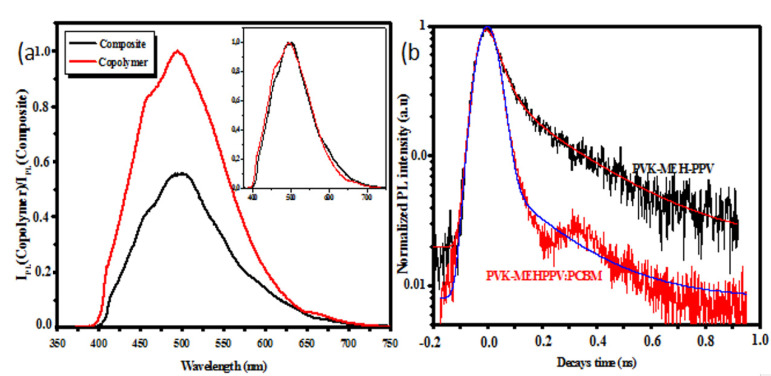
(**a**) Stationary and (**b**) time-resolved photoluminescence spectra of the copolymer and composite.

Although the PL spectra of the PVK-MEH-PPV copolymer [23] and the composite were obtained under the same experimental conditions at RT, there were no major differences between them compared to the two normalized PL spectra (Figure 5a).

However, the difference in the ratio of the I495/I460 bands of 1.2 in the copolymer to 1.4 in the case of the composite should be noted; it could be explained by the charge transfer population from the 460 to 495 nm peaks. This could have been the result of a new electronic reorganization in the copolymer matrix caused by the presence of PCBM. The charge transfer from the copolymer to the PCBM was confirmed by quenching the intensity of the photoluminescence passing from the copolymer (λ_emission_ = 495 nm, *I_d_* = 1) to the composite (λ_emission_ = 496 nm, *I_d/a_* = 0.553), where *I_d/a_* and I_d_ are the integrated PL intensities of the donor in the presence and absence of the acceptor, respectively. This implies that PCBM acts like an electron acceptor that separates the excitons and leads to the dampening of the photoluminescence and the charge transfer. This indicates good PCBM percolation in the copolymer matrix, leading to a faster photo-induced electron transfer. That means that PCBM can lock as an electron trap of photogenerated excitons, manifesting the charge transfer phenomena. The value of the charge transfer efficiency was found to be 0.447 for the composite (PVK-MEH-PPV:PCBM), taking into account the pursuant relation [32]:

(1) $$\eta_{T}=1-\frac{I_{d/a}}{I_{d}}$$

where, $\eta_{T}$ is the charge transfer efficiency.

Figure 5b shows the normalized TR-PL of our previous PVK-MEH-PPV copolymer [23] and the composite (PVK-MEH-PPV:PCBM). We used 400 nm as the excitation energy and the spectra were integrated between 350 and 750 nm. The decay times of the normalized-intensity PL were simulated with two exponential decays, the first decay being faster than the second. Subsequently, these two decays were calculated by taking into consideration the contribution of the Gaussian time-dependence function G(t) of the laser pulse, as indicated in previous work [23,24,25,26]. Table 1 shows the weights P_1_ and P_2_, the decay times τ_1_ and τ_2_ for each exponential and the average decay time τ_mean_ characteristic of the PL lifetime. The composite exhibited a decrease in both lifetimes from 0.040 ns to 0.015 ns for τ_1_ and from 0.270 ns to 0.209 ns for τ_2_ when we compared it to the copolymer. Consequently, the average decay time was lower when comparing the composite to the copolymer. This can be clarified as follows: the PCBM introduced into the PVK-MEH-PPV matrix trapped the electrons of photogenerated excitons, leaving holes in the PVK-MEH-PPV chains, which created a quicker decay PL. This leads to the conclusion that PCBM acts as a quencher, separating and favoring the non-radiative pathway of photogenerated excitons. This may be useful for the collection of charge in the electrodes if the present composite is used as an active layer in photovoltaic cells.

The photogenerated charge fractions in the largest/shortest weighted segments represented 56.7% and 43.3%, respectively, for the copolymer. In the case of the composite, these contributions were respectively equal to 85.4% and 14.5%. Moreover, the reduction in the populations of photo-generated fillers in the long segments led to a lifelong strength for the copolymer lifetime. However, we can propose that the existence of PCBM particles induced shorter conjugate segments, resulting in a short decay time. For TR-PL, the value of the charge transfer efficiency was found to be 0.747 for the composite (PVK-MEH-PPV:PCBM), taking into account the pursuant relation [32]:

(2) $$\eta_{T}=1-\frac{\tau_{d/a}}{\tau_{d}}$$

where *τ_d/a_* and *τ_d_* are the average lifetimes of the photogenerated charge of the copolymer in the presence and absence of PCBM, sequentially.

### 3.5. Theoretical Part

#### 3.5.1. Electronic Properties and Energy Diagram of the Composite

**Figure 6:**
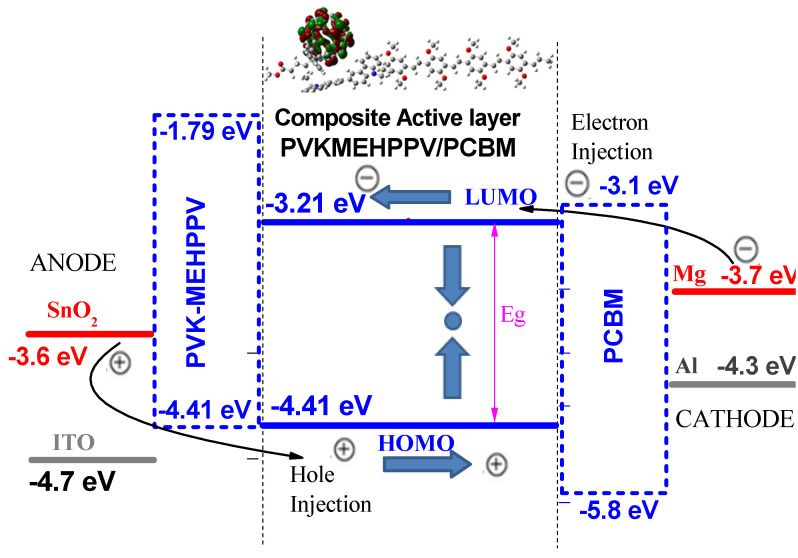
Frontier molecular orbitals of HOMO/LUMO and schematic energy diagram of the composite with different electrodes.

It is important to note that, in this theoretical segment, all the calculations were performed using the structure of the model of the previous copolymer PVK-MEH-PPV [23] and PCBM (Figure 6). First, we optimized a PCBM compound, to which we added the optimized PVK-MEH-PPV model structure, placing the PCBM with the copolymer using Gauss view software.

The composite (PVK-MEH-PPV:PCBM) showed a low band gap of approximately 1.2 eV with HOMO and LUMO energy levels of about −4.41 and −3.21 eV, respectively. On the one hand, the frontier molecular orbitals (MOs) of the HOMO and LUMO levels of the composite in the ground state were designed (Figure 6). The MOs of the LUMO and HOMO levels were localized to the acceptor (PCBM) and the donor (copolymer), respectively. On the other hand, the charge transfer between the donor and acceptor groups was further clarified by electronic analysis when the binding energy was calculated to be −0.006 eV, taking into account the following relation [33]:

[(E_composite_ − (E_copolymer_ + E_PCBM_)] (3)

The V_OC_ of the modeling blend structure of a PVK-MEH-PPV/PCBM-based solar cell would be a high open-circuit voltage of 1 eV, which would produce an effective solar cell device.

V_OC_ = 1/e|E(HOMO)donor| − | E(LUMO)acceptor|− 0.3;

where e is the elementary charge and the value of 0.3 is an empirical factor.

In order to present the composite-based photovoltaic cell energy diagram model (PVK-MEH-PPV:PCBM), different anodes and cathodes (ITO, SnO_2_, Mg, Ca, Al, Ag, Au) were tested to select the best-performing one and to avoid the interface problem.

The output work and the energies of the HOMO and LUMO levels of the active layer levels should be small in order to easily collect the electrons and holes at the electrodes. For this purpose, Mg and SnO_2_ were chosen to be used as the cathode and anode, respectively (Figure 6). However, it is clear that the LUMO level of the composite (−3.31 eV) depends strongly on the acceptor (PCBM (−3.38 eV)), while the HOMO level can be estimated to have the same value as the donor (copolymer).

#### 3.5.2. Theoretical Optical Absorption

All the electronic transitions of the copolymer and composite spectra are illustrated in Table 2.

**Figure 7:**
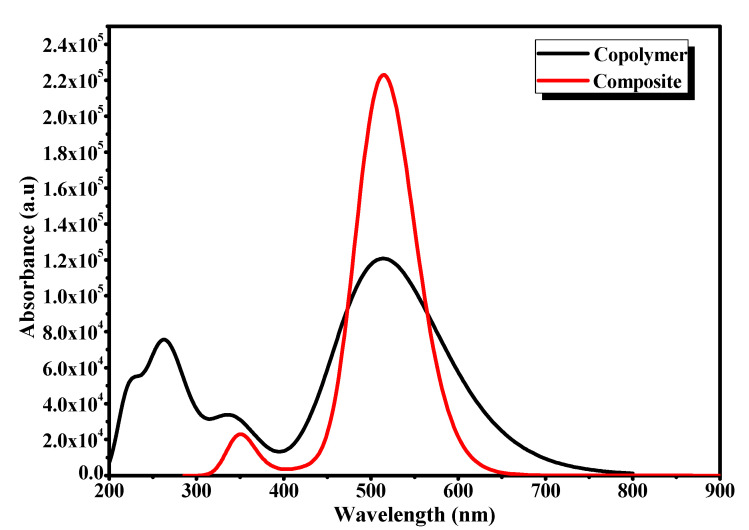
Theoretical optical absorption spectra of the model structures of the copolymer and composite calculated by TD-DFT (B3LYP).

By comparing the theoretical absorption spectra of the copolymer and the composite (Figure 7), we can note that some bands disappeared (229 and 264 nm) and others showed slight changes in positions or intensities. More precisely, the band in the composite spectra located at 350 nm was decreased and red-shifted compared to that of the copolymer. On the other hand, the band located at 515 nm was enhanced. The appearance of this band with strong intensity in calculated absorption spectra compared to a very weak intensity in the experimental spectra could have been related to the VdW interaction between PCBM and the PVK-MEH-PPV side chain, as well as PVK units in the backbone. In contrast, it could also have been related to the PCBM position facing the copolymer structure and the absence of interchain interaction; this can deform the conjugated backbone and dramatically change the spectra.

All of these changes relate to the intermolecular charge transfer between the donor and acceptor [34,35,36]. The charge transfer can be explained in the ground state by the variation of the total copolymer charge density from 0.330 e in the absence of the PCBM to 0.331 e in the presence of PCBM.

The optical gap was determined with the λ_onset_ method [6] and was calculated to be 2.07 eV from the theoretical absorption spectra using the following equation:

E_g_ = 1240/λ_onset_ (4)

It is important to recall that the calculations were done based on one isolated chain model structure of the copolymer with one PCBM molecule. Therefore, it is reasonable to conclude that the difference between the experimental and calculated gap was due to the absence of interchain interaction and the small system modeling and charge transfer accrued in the composite with a large number of PCBM molecules in the PVK-MEH-PPV matrix. The difference can be also attributed to the gas phase (theoretical calculations) and to the solid-state (experimental) effect.

**Figure 8:**
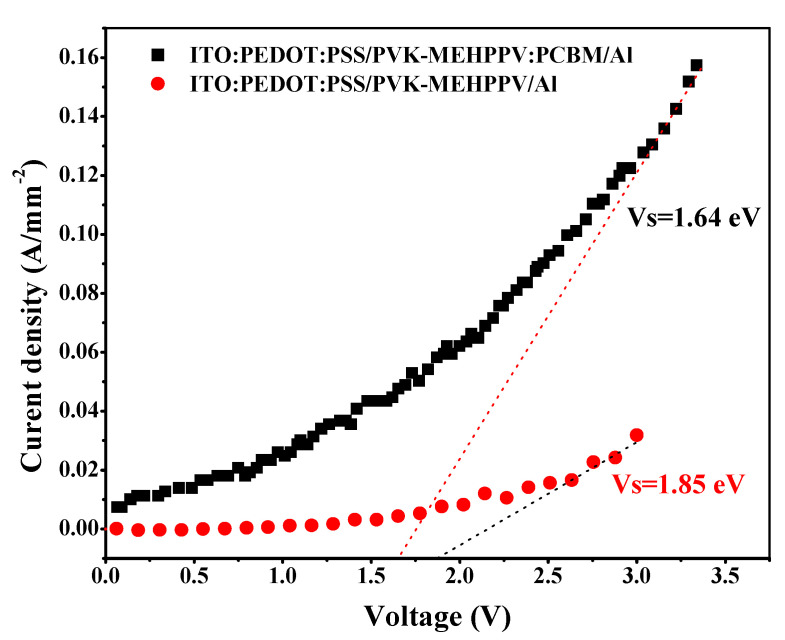
The J-V characteristics of the ITO/PEDOT:PSS/PVK-MEH-PPV:PCBM/Al structure.

In order to confirm that our structure is a good candidate for PV applications, Figure 8 shows the diode behavior for the ITO/PEDOT:PSS/PVK-MEH-PPV:PCBM/Al structure.

The threshold voltage appears to be enhanced compared to ITO/PEDOT:PSS/PVK-MEH-PPV/Al, which confirms that charge transfer occurs between the donors (copolymer) and PCBM as the acceptor. The charges are then strongly transported to the electrodes. The enhancement of Vs is related to the reduction of the barrier height.

In the end, the PVK-MEH-PPV copolymer seems to be a good material with a simple method of synthesis and good reproducibility, which includes interesting optoelectronic properties, in particular the intermolecular charge transfer. The optimal properties of the resulting copolymer were derived from the combination of PVK and MEHPPV homopolymers. On the other hand, the thermally stable composite-based PCBM and copolymer have the same simplicity and reproducibility in their synthesis as those of the copolymer. Therefore, using the PCBM/polymer as an active layer in the future may prove to be advantageous if cost, stability and efficiency issues can be well balanced. The real advantages of this composite are its applications for lightweight and flexible devices and the fact that it can be processed in solution with more thin film.

This study aims to provide guidance for researchers developing active materials and functional composites in photovoltaic cells.

It is important to note that this study involved some limitations as follows: For the method of characterization, we did not utilize the dark current and photocurrent measurements or the quantitative photoconversion analysis. With regard to the materials, as is well known, polymers are degradable materials with low charge collection efficiency. Furthermore, we must not forget the complicated purification process and the percolation phenomenon of the PCBM in the copolymer matrix, which influences the charge transport.

## 4. Conclusions

In summary, the study of the properties of the PVK-MEH-PPV:PCBM composite were examined and their characterization carried out. The functionalization, stacking interaction and charge transfer were investigated using various experimental analyses, such as optical absorption, photoluminescence and time-resolved photoluminescence. It was shown that the addition of PCBM to the copolymer matrix was accompanied by charge transfer, photoluminescence quenching and a decrease of band-gap energy. This charge transfer was confirmed by quenching with Uv-Vis and PL spectra and by the short mean decay time in TR-PL. In addition, density functional theory (DFT) was used to study the charge transfer, especially in molecular orbitals and electronic structures, and it confirmed the interaction and charge transfer between donor and acceptor molecules. Overall, it can be concluded that this new composite (PVK-MEH-PPV:PCBM) can potentially be applied as an active material in photovoltaic cells. Our study indicates that the addition of PCBM to the PVK-MEH-PPV copolymer effectively adapts the band-gap energy. Sadly, the photoconductivity and quantitative photoconversion measurements of the resulting composite were not accessible in our laboratory at our university.

## Figures and Tables

**Table 1:** Decay times and parameters were obtained from the analysis of the experimental PL decay for the copolymer and the composite.

	P_1_ (%)	P_2_ (%)	τ_1_ (ns)	τ_2_ (ns)	τ_mean_ (ns)
Copolymer [20]	56.7	43.3	0.04	0.27	0.17
Composite	85.4	14.6	0.015	0.209	0.043

**Table 2:** Electronic transitions of the theoretical optical absorption of the copolymer and composite.

TD-DFT/3-21G *		λ_max_ (nm)	Energy (eV)	Oscillator Strength (f)	Assignment
Composite	T1	515	2.41	0.8226	H-13→L+0 (47%)H-0→L+5 (+26%)
	T2	350	3.45	0.2590	H-1→L+8 (83%)
Copolymer	T1	514.3	2.41	2.9702	H-0→L+0 (+99%)
	T2	338.3	3.66	0.4809	H-8→L+0 (+73%)
	T3	264.3	37.8	0.0108	H-3→L+2 (+82%)
	T4	229.6	5.40	0.0137	H-18→L+0 (+57%)

## Data Availability

Exclude this statement if the study did not report any data.
